# Exosome-inflammasome crosstalk and their roles in inflammatory responses

**DOI:** 10.7150/thno.54004

**Published:** 2021-03-04

**Authors:** Chadanat Noonin, Visith Thongboonkerd

**Affiliations:** Medical Proteomics Unit, Office for Research and Development, Faculty of Medicine Siriraj Hospital, Mahidol University, Bangkok 10700, Thailand.

**Keywords:** caspase-1, IL-1β, IL-18, inflammatory disease, miRNAs, NLRP1, proteins, therapeutics

## Abstract

Inflammasome is a complex of multiple proteins found in cytoplasm of the cells activated by infectious and/or non-infectious stimuli. This complex involves caspase-1 activation, leading to unconventional secretion of interleukin-1β (IL-1β) and IL-18 and inflammatory cascade. Exosome is the nanoscale membrane-bound extracellular vesicle that plays significant roles in intercellular communications by carrying bioactive molecules, e.g., proteins, RNAs, microRNAs (miRNAs), DNAs, from one cell to the others. In this review, we provide the update information on the crosstalk between exosome and inflammasome and their roles in inflammatory responses. The effects of inflammasome activation on exosomal secretion are summarized. On the other hand, the (dual) effects of exosomes on inhibiting and promoting inflammasome activation are discussed. Finally, perspectives on therapeutic roles of exosomes in human diseases and future direction of the research on exosome-inflammasome crosstalk are provided.

## Introduction

Inflammation is an immune response that can be triggered by infectious and/or non-infectious stimuli, e.g., cell damage, physical injury, toxins. This response is primarily triggered to cope with such harmful stimuli and to promote repairing process 1. However, excessive or prolonged inflammation has deleterious effects that finally lead to tissue damage as observed in many inflammatory diseases, e.g., rheumatoid arthritis, colitis, asthma 2. Inflammation is initiated after host proteins, collectively known as pattern recognition receptors (PRRs), bind to exogenous molecules called pathogen-associated molecular patterns (PAMPs) (leading to infection-related inflammation) or to endogenous ones called damage-associated molecular patterns (DAMPs) (leading to non-infection-induced inflammation). These bindings activate downstream intracellular signaling pathways, leading to production and secretion of inflammatory cytokines, which can exert pro-inflammatory or anti-inflammatory effects based on type of the stimuli and stage of inflammatory processes 1.

Known PRRs include: 1) Toll-like receptors (TLRs), 2) C-type lectin receptors (CLRs), 3) retinoic acid-inducible gene (RIG)-I-like receptors (RLRs), 4) nucleotide-binding oligomerization domain (NOD)-like receptors (NLRs), 5) absent in melanoma-2 (AIM2)-like receptors (ALRs), and 6) pyrin 3. TLRs and CLRs are membrane proteins responsible for recognition of extracellular stimuli, whereas the rest are cytoplasmic receptors that recognize intracellular ligands. Pyrin protein and some members of NLRs and ALRs are responsible for activation of inflammasome, which is a cytoplasmic multi-protein complex that promotes caspase-1-dependent secretion of interleukin-1β (IL-1β) and IL-18 and pyroptosis 3.

The term “inflammasome” was first described by Martinon and colleagues in 2002 as a large protein complex assembled upon recognition of PAMPs or DAMPs by PRRs 4. Among several proteins recruited into this complex, the last one is pro-caspase-1 that is then activated to caspase-1, resulting in unconventional secretion of IL-1β and IL-18, which are the cytokines lacking signal peptide 4, 5. Interestingly, various PRRs cause generation of different inflammasome complexes 6. One of the possible routes that IL-1β and IL-18 are unconventionally secreted from the cells is via releasing of endosomal vesicles, namely exosomes, because IL-1β and IL-18 are found in the released exosomes 5, 7-9.

Exosomes are the nanoscale membrane-bound extracellular vesicles (EVs) that are secreted by most of eukaryotic cells and contain bioactive molecules, e.g., proteins, RNAs, microRNAs (miRNAs), DNAs, metabolites, which can be transferred from one cell to the others 10-13. Therefore, exosomes have important roles in the intercellular communications by transferring different messages between cells in the form of bioactive molecules they carry 14. Depending on molecules packed into exosomes, they can perform various tasks, e.g., promoting cell proliferation and migration 15, lowering oxidative stress and apoptosis 16, activating cytokine secretion 17, and inhibiting or stimulating inflammasome activation (discussed later in this review).

**Figure 1:**
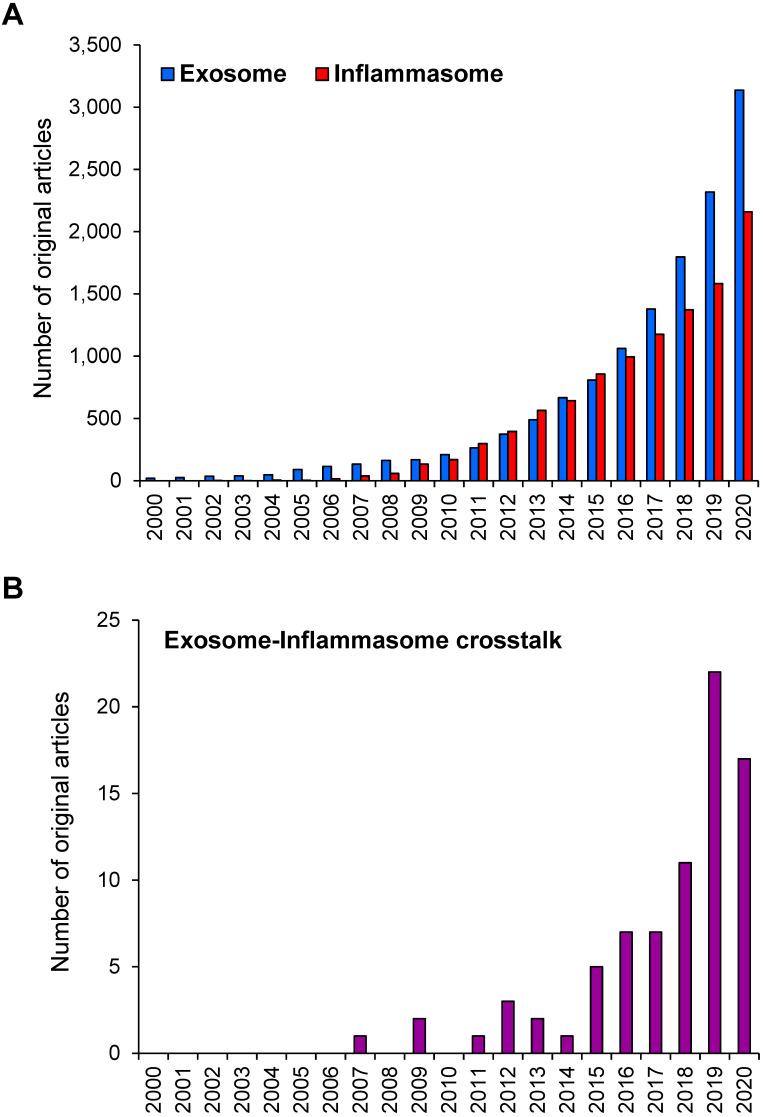
** Number of the original articles on exosome and/or inflammasome in PubMed. (A)** Number of the original articles using the keyword “exosome” OR “inflammasome”. **(B)** Number of the original articles using the keyword “exosome AND inflammasome”. Note that the reviews and other non-original articles were excluded.

During the past two decades, research on exosome and inflammasome has gained a worldwide attention. Based on the PubMed search using the keyword “exosome” OR “inflammasome”, numbers of the original articles reported on the studies in these two areas have been increasing exponentially since 2000 (reviews and other non-original articles were excluded) (**Figure 1A**). Using the term “exosome AND inflammasome”, number of the original articles reporting the exosome-inflammasome crosstalk has been also increasing (**Figure 1B**). Although the latter number is relatively small compared with that of each field, these data indicate the emerging roles of exosome-inflammasome crosstalk in biomedical sciences. In this review, we provide the update information on the crosstalk between exosome and inflammasome as well as their roles in inflammatory responses. Some studies have demonstrated that inflammasome activation can regulate the release of exosomes. On the other hand, other lines of evidence have shown that exosomes are the upstream regulators for inflammasome activation.

## An overview of inflammation

Common symptoms of local inflammation are swelling, redness, heat and pain resulting from pro-inflammatory cytokines released by immune cells in response to infection, toxicity, tissue injury, etc. These pro-inflammatory cytokines play crucial roles in recruiting immune cells to the injured sites, stimulating cell proliferation, and inducing apoptosis, all of which finally lead to destruction of the foreign bodies, toxins or microbes, elimination of the damaged cells, and restoration of homeostasis 2, 18. Upon triggering of PRRs by PAMPs or DAMPs, intracellular inflammatory signaling pathways are activated accompanied with upregulation or downregulation of several genes/proteins and chemokine secretion 18, 19. These inflammatory signaling pathways include nuclear factor kappa-B (NF-κB), Janus kinase (JAK)-signal transducer and activator of transcription (STAT), and mitogen-activated protein kinase (MAPK) pathways. Among them, NF-κB is the key pathway responding to PAMPs or DAMPs, while JAK-STAT and MAPK pathways respond primarily to cell stress, cytokines, or hormones 18, 20.

Interestingly, NF-κB pathway has a direct link with inflammasome, because *IL1B* gene encoding IL-1β is one of the targets for NF-κB transcription factor 21. However, activation of NF-κB pathway alone is not sufficient to stimulate inflammasome-dependent IL-1β secretion (discussed in more detail in the next section). Under unstimulated condition, NF-κB binds to its inhibitor (IκB) and remains inactive in the cytoplasm. Once stimulated, NF-κB is released from IκB and then activated, followed by translocation into nucleus to bind and modify its targets, e.g., tumor necrosis factor α (TNFα), IL-1β, IL-6, IL-8, IL-18, C-X-C motif chemokine 10 1, 22, 23. Receptors located upstream of NF-κB activation are TLRs, TNF receptor (TNFR), T-cell receptor, B-cell receptor, and IL-1 receptor (IL-1R) 22.

## Inflammasome complex formation and activation

Inflammasome is a complex of multiple proteins found in cytoplasm of the activated cells. Formation of this complex is necessary for caspase-1 activation that leads to conversion of pro-IL-1β and pro-IL-18 to IL-1β and IL-18, respectively, both of which are the active forms that are then secreted out of the cells 24. In addition, caspase-1 can mediate gasdermin D-induced pyroptosis, which is an inflammatory form of the programed cell death 25. The inflammasome complex was initially identified by a study that aimed to investigate mechanism of pro-inflammatory caspase activation 4. Components in this complex that were initially identified include NLRP1 (a protein in NLR family), apoptosis-associated speck-like protein containing a caspase recruitment domain (ASC), and caspase-1 4. The same research group later reported that NLRP2 and NLRP3 can also form the inflammasome complex with ASC and caspase-1 (but not caspase-5) 26. Inflammasome is a part of innate immune system, which can be activated by infection or endogenous danger signals such as oxidative stress, self-DNA, and ATP 6, 27-31. Since the output of inflammasome activation is the secretion of pro-inflammatory cytokines, inflammasome is thus associated with inflammation 1. Dysregulation of inflammasome activation is the pathophysiologic mechanism underlying many inflammatory diseases, such as multiple sclerosis, atherosclerosis, Parkinson's disease, and Alzheimer's disease 27. Inflammasome can be activated through two different pathways - canonical and non-canonical activation pathways.

### Canonical inflammasome activation

Canonical activation of the inflammasome complex is initiated by cytoplasmic recognition of PAMPs or DAMPs by PRRs 25. Such binding activates the receptors, resulting in a recruitment of ASC, which acts as an adaptor protein, to the complex. Pro-caspase-1 participates in the complex by binding to the caspase recruitment domain of ASC. Activation of pro-caspase-1 to caspase-1 leads to processing of pro‑IL‑1β and pro‑IL‑18, and then secretion of their mature forms. In addition, active caspase-1 cleaves gasdermin D to generate its N-terminal products, which then translocate to inner leaflet of the cell membrane 25. The N-terminal fragments of gasdermin D can bind phosphatidylinositol 4-phosphate, phosphatidylinositol 4,5-bisphosphate, and/or phosphatidylserine to form an oligomer. Such oligomerization causes pore formation in the cell membrane, leading to pyroptosis, which is characterized by cell swelling and membrane rupture 32.

The common PRRs in the canonical inflammasome pathway include NLRP1 4, NLRP3 33, 34, NLR family apoptosis inhibitory protein (NAIP)-NLRC4 35, 36, pyrin 37, 38, and AIM2 28, 39. These PRRs recognize different PAMPs and DAMPs. Among them, NLRP3 is most frequently investigated and has the most diverse activators 3, 6, 30, 40, 41. Unlike other PRRs, NLRP3 and pyrin can be activated without a direct contact with their activators. NLRP3 activators, such as nigericin, ATP, gramicidin, and bacterial aerolysin and α-hemolysin, can induce NLRP3 activation through K^+^ efflux 42. Amyloid-β, another NLRP3 activator, triggers the lysosomal release of cathepsin B, which then induces NLRP3 activation 43. For pyrin, toxins released from the endocytosed bacteria can inactivate Rho GTPase, and such inactivation can induce the pyrin inflammasome activation 44.

### Non-canonical inflammasome activation

For non-canonical pathway, activation of inflammasome involves function of species-specific caspases (human caspase-4, human caspase-5, or mouse caspase-11). In this pathway, lipopolysaccharide (LPS) from bacterial cells or outer membrane vesicles is endocytosed into the host cells. After LPS is released from the endosomes, it is recognized by human caspase-4, human caspase-5, or mouse caspase-11. These caspases then act as the cytosolic PRRs to bind the intracellular LPS to get activated 6, 45-47. The activated human caspase-4, human caspase-5, or mouse caspase-11 then cleaves gasdermin D, resulting in pyroptosis 25, 47, 48. In addition, by unknown mechanism, mouse caspase-11 can stimulate NLRP3 inflammasome formation, leading to secretion of the active forms of IL-1β and IL-18 6, 49.

### Two signals are required for inflammasome-dependent IL-1β secretion

**Figure 2:**
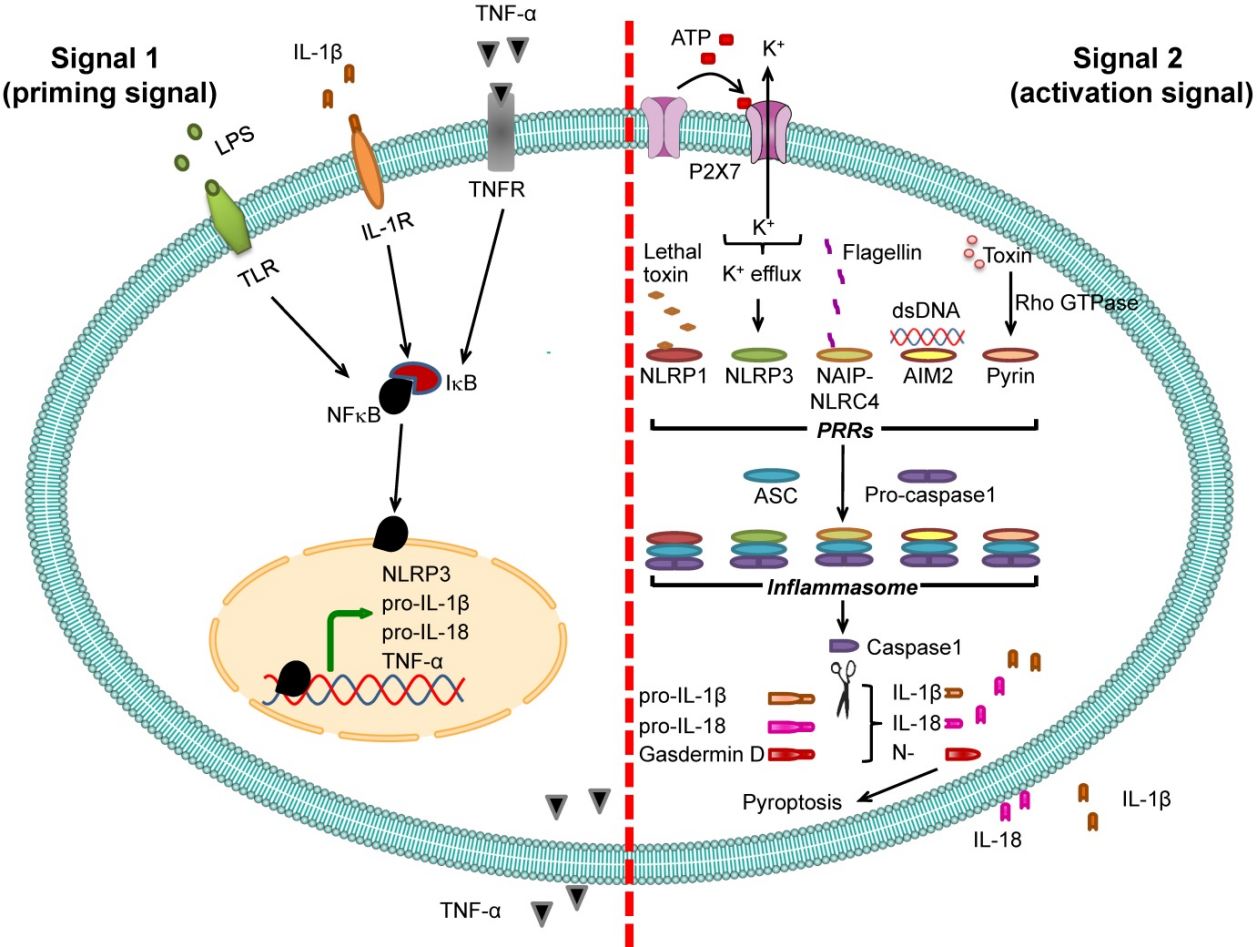
** Inflammasome activation.** Two signals are required for inflammasome activation. The first is “signal 1” or “priming signal”, which acts through NF-κB signaling pathway. The priming signal starts with binding of PAMPs (e.g., LPS) or DAMPs (e.g., heat shock proteins) to TLR, IL-1β to IL-1R, or TNF-α to TNFR (see more details in **Table 1**). Such receptor binding subsequently activates NF-κB signaling pathway by dissociating NF-κB from its inhibitor IκB and translocating NF-κB into the nucleus to upregulate expression of its target genes encoding NLRP3, pro-IL-1β, pro-IL-18, and TNF-α. The second is “signal 2” or “activation signal”, which acts through the induction of PRRs by various PAMPs or DAMPs (see more details in **Table 2**), resulting in recruitment of ASC and pro-caspase-1 to form inflammasome complex with such PRRs. Pro-caspase-1 is then cleaved to caspase-1 that subsequently converts pro-IL-1β and pro-IL-18 to IL-1β and IL-18, respectively, leading to secretion of IL-1β and IL-18. From both signals, the secreted IL-1β and TNF-α can potentiate activation of NF-κB signaling pathway and in turn increase their own production. In addition, caspase-1 cleaves gasdermin D and the N-terminal fragments of gasdermin D cause pyroptosis, an inflammatory form of cell death.

Pro-IL-1β expression is relatively low under normal physiologic condition but is markedly increased by infection 50. Promoter region of the gene encoding pro-IL-1β (*IL1B*) contains binding site for NF-κB transcription factor, and binding of NF-κB to this region activates pro-IL-1β transcription. Therefore, expression of pro-IL-1β is under regulation of NF-κB 21, 51. Molecules that stimulate NF-κB binding to pro-IL-1β gene (*IL1B*) promoter are called “signal 1” or “priming signal” (**Table 1**). They are the extracellular molecules recognized by cell membrane PRRs such as TLRs. On the other hand, the molecules that subsequently activate pro-IL-1β after binding of NF-κB are called “signal 2” or “activation signal” and play essential roles in the inflammasome activation (**Table 2**). Therefore, inflammasome activation firstly requires signal 1 that activates NF-κB and its translocation into nucleus in order to turn on pro-IL-1β gene (*IL1B*) expression and secondly needs signal 2, such as ATP and K^+^ efflux as well as other several PAMPs and DAMPs, to stimulate assembly of inflammasome in order to convert pro-IL-1β to active IL-1β, and to induce its secretion 23, 52 (**Figure 2**).

As mentioned above that ligand-bound IL-1R causes NF-κB activation, the secreted IL-1β further enhances its own expression and inflammatory response through NF-κB pathway 23. In the case of NLRP3-containing inflammasome, NF-κB is important not only for triggering pro-IL-1β expression but also for stimulating NLRP3 expression through binding of the first signal to TLR4 53. Stimulation of macrophages with only ATP or nigericin (signal 2) is adequate to initiate inflammasome assembly and to activate caspase-1, when macrophages are overexpressed with NLRP3 54. The requirement of signal 1-activated NF-κB to upregulate IL-1β, IL-18 and NLRP3 expression therefore indicates the coordination of inflammasome dependent and independent inflammation for proper inflammatory response.

Any molecules that interfere with either signal 1 or signal 2 can alter inflammasome activation. An endogenous molecule GTPase Rab1a has been reported to play role in regulating plasma membrane localization of TLR4, and dysfunction of Rab1a prevents NF-κB activation, thereby inhibiting NLRP3 inflammasome activation 55. M013 protein from Myxoma virus has been shown to prevent nuclear translocation of NF-κB and, as a result, IL-1β secretion is reduced 56. In addition, treating the cells with a specific inhibitor of NF-κB can suppress pro-IL-1β transcription upon activation 57. Moreover, betaine inhibits inflammasome activation by interrupting both NF-κB translocation and inflammasome assembly 58.

A remark here is that the mechanisms for inflammasome activation discussed above are just some of the common phenomena that generally occur. It should be noted that various types of the cells may express differential PRRs 59. Moreover, different cells, e.g., macrophages, monocytes, neutrophils and endothelial cells, may use different PRRs and upstream signal pathways of NF-κB activation for the inflammasome activation (for more details please see 60).

## Exosome: biogenesis, biology, function and regulation

Exosome, with a size of approximately 40-150 nm, is one among the three types of membrane-bound EVs, whereas the other two are microvesicle (membrane shedding vesicle, with a size of 150-1,000 nm) and apoptotic body (with a size of 1-5 µm) 11, 61. In this review, the term “EVs” is used when the cited references refer to all membrane-bound secreted vesicles including exosomes, whereas the term “exosomes” is used when the cited references really mean exosomes in their studies. Exosomes are originated from late endosomes of which membrane becomes inward budding to form intraluminal vesicles (ILVs) within endosomal compartment 61. The late endosomes containing ILVs, namely multivesicular bodies (MVBs) (also called multivesicular endosomes), then traffic to and fuse with plasma membrane of the cells in order to release such ILVs 62. Membrane trafficking of the MVBs is regulated by proteins such as small GTPase Rab27, Rab27b and Rab interacting lysosomal protein (RILP), which is a Rab7 trafficking adaptor protein 63, 64. After release, these ILVs are called exosomes, which can be isolated by ultracentrifugation at 100,000-200,000 ×*g*. Several proteins have been reported to serve as the markers for exosomes, e.g., CD63, CD9, CD81, ALIX (apoptosis linked gene 2 (ALG-2) interacting protein X), and TSG101 (tumor susceptibility gene 101) 11, 61. The presence of EVs and exosomes in biological fluids, e.g., blood 65, 66, cerebrospinal fluid 67, amniotic fluid 68, saliva 65, 68, urine 68, and breast milk 65, has been reported and their increase in these biological fluids or conditioned medium is correlated with inflammation 69-71.

As mentioned above, exosomes are composed of various proteins, RNAs, miRNAs, DNAs, lipids, and metabolites. Amounts of these bioactive molecules carried by exosomes vary, depending on type of the cells releasing them 11 and cell differentiation stage 72. For example, muscle cells in various differentiation stages release exosomes containing differential protein contents (or proteome) 72. Furthermore, the amounts of several molecules carried by exosomes are altered under specific conditions. For example, exosomes released from virus-infected cells contain high amount of the viral proteins 73, 74. Cancer cells treated with chemotherapeutic compound secrete exosomes rich with DNA contents 10. Exosomes derived from LPS-treated macrophages comprise greater amount of several proteins, especially cytokines, compared with the control 75. Exosomes released from macrophages treated with calcium oxalate monohydrate (COM) contain higher levels of vimentin and annexin A2 but lower levels of heat shock protein 90β and calreticulin 76, 77. Under abnormal conditions, EVs can carry a high level of inflammasome components such as those found in EVs derived from sera of stroke patients 66, traumatic brain injury patients 78, and reproductively senescent women 79. In addition, hypoxic and starved mesenchymal stem cells (MSCs) can secrete exosomes containing a high level of metabolites with immunomodulatory activities to induce T-cell activation and macrophage polarization 9.

The molecules carried by exosomes play important roles in transferring messages from the releasing cells to the others. These messages allow the other cells to perceive biological events around them and also guide them how to respond. For example, once exosomes derived from macrophages collected from patients with glioblastoma multiforme (GBM) are taken up by the GBM cells, an miRNA (miR-21) residing in these exosomes can inhibit expression of a tumor suppressor gene* PDCD4* (programmed cell death 4) in these cells, allowing the cells to grow further 80. In addition, these miR-21-containing exosomes induce the recipient GBM cells to resist to a chemotherapy with temozolomide 80. Transport of miR-1246 by exosomes from human umbilical cord blood mesenchymal stem cells (hUCBMSCs) to the liver reduces inflammation and liver damage caused by hepatic ischemia/reperfusion injury 81. Exosomes containing miR-155 are able to promote macrophage recruitment into the lungs of naive mice and also increase macrophage proliferation *in vitro* 70. Moreover, the non-infected cells can be affected by the virus infection via viral protein-carrying exosomes secreted by the nearby virus-infected cells 74, 82. Specifically, Epstein‐Barr virus (EBV)-infected B-cells and nasopharyngeal carcinoma (NPC) cell line stably expressed an EBV protein namely latent membrane protein 1 (LMP1) can secrete EVs and exosomes carrying this viral protein. The uptake of these LMP1-containing exosomes by the non-infected B-cells and NPC cells enhances B-cell proliferation, tumor growth and radioresistance of the NPC cells 74, 82. In a kidney stone model, exosomes derived from COM-treated macrophages enhance T-cell migration and macrophage phagocytic activity 76. These enhancements are most likely due to the upregulation of vimentin in exosomes derived from the COM-treated macrophages because T-cell migration and macrophage phagocytic activity can be suppressed by knockdown of vimentin in the COM-treated macrophages 76.

Taken together, the aforementioned data indicate that the process, in which various biomolecules are packed into the exosomes, is considerably cell-specific and selective. A protein namely KRAS (Kirsten rat sarcoma viral oncogene homolog) has been reported to participate in sorting miRNAs into exosomes, because differences of miRNAs inside the exosomes secreted from different colorectal cancer cell lines are correlated with mutations of the *KRAS* gene 83. Specific packing of miR-143 into exosomes of endothelial cells under shear stress has been shown to be regulated by GTPase Rab7a and Rab27b 84. The packing of miR-155 into exosomes derive from hepatitis C virus (HCV)-infected hepatoma cells is controlled by RILP 64. In addition, CD63 participates in loading of LMP1 into exosomes of the EBV-infected cells 85. Furthermore, different isoforms of neutral sphingomyelinase play roles in packing normal soluble or misfolded forms of cellular prion protein into exosomes secreted from mouse hypothalamic neuronal cell line 86. Although there are some reports on proteins responsible for selective packing of molecules into EVs, which also include exosomes, a mechanism underlining this process remains unclear 14. Regarding the secretion of exosomes, various factors have been reported to stimulate exosomal secretion. Acidic environment has been demonstrated to enhance exosomal release from metastatic human melanoma cells 87. Elevation of intracellular calcium level is another factor triggering the secretion of exosomes 88. In concordance, binding of extracellular ATP to P2X7 receptor, which causes an increase of cytoplasmic calcium level, also enhances the secretion of exosomes and EVs 89. Furthermore, hypoxic condition enhances the secretion of exosomes from breast cancer cell lines 90. As mentioned above that exosomal amount in biological fluids is related to inflammatory condition, the activation of immune pathway, such as LPS-stimulated TLR4 91 or inflammasome may also enhance exosomal secretion (discussed in more detail in the next section).

## Exosome-inflammasome crosstalk

The crosstalk between exosome and inflammasome has been documented with increasing evidence during recent years. Interestingly, some studies have demonstrated that inflammasome activation can regulate the release of exosomes. On the other hand, exosomes are the upstream regulator for inflammasome activation. Most of the latter studies have elucidated the effects of exosomes on inflammasome activation by utilizing exosomes isolated from either biological fluids or cell culture media. The results have demonstrated that exosomes exert the dual effects. Several studies have shown their inhibitor effects on inflammasome activation, whereas other lines of evidence have shown the opposite data indicating the positive correlation between exosomal release and inflammasome activation. We thus summarize all the results obtained from these studies (**Tables 3 & 4**) and discuss below.

### Effects of inflammasome activation on exosomal secretion

Inflammasome activation had been previously assumed to be an enhancer for EVs secretion because several NLRP3 inflammasome activators can also stimulate EVs secretion 92. Bone marrow-derived macrophages (BMDMs) primed with IFN-γ and LPS secrete exosomes and microvesicles containing major histocompatibility complex (MHC) class II after exposure to ATP 93. Secretion of the MHC II-containing exosomes, but not the MHC II-containing microvesicles, has been shown to be inflammasome-dependent because BMDMs obtained from mice lacking ASC or NLRP3 fail to secrete these exosomes after IFN-γ/LPS priming followed by ATP treatment 93. In addition, synovial fibroblasts treated with IL-1β secrete significantly greater amount of exosomes as compared with the untreated control cells 69. IL-1β may stimulate exosomal secretion via activation of NF-κB because it is one of the NF-κB activators. NF-κB upregulates expression of LAMP-2 and Rab34, which are involved in membrane trafficking 94. NF-κB also facilitates membrane trafficking of glucose transporter-1 via AKT activation 95. Recently, a direct evidence for inflammasome-dependent exosomal secretion has been reported 64. HCV infection or induction with LPS followed by ATP can trigger NLRP3 inflammasome activation in Huh-7.5 cells. Also, NLRP3 overexpression in the uninfected cells increases exosomal secretion. Interestingly, subsequent caspase-1-dependent cleavage of RILP is responsible for the increase in exosomal secretion and packaging of specific miRNAs inside 64. In addition, fragile X mental retardation 1 (FMR1) protein together with endosomal sorting complex required for transport (ESCRT) plays roles in packaging of specific miRNAs with FMR1-binding motif into the exosomes 64.

By contrast, another study has reported that inflammasome activation may not be required for exosomal secretion. ATP is a known activator (signal 2) of NLRP3 inflammasome complex to induce NLRP3 inflammasome assembly 29 (**Table 2**). Together with pre-treatment of LPS, IL-1β secretion is enhanced 89. Interestingly, ATP alone is inadequate to stimulate IL-1β secretion but can strongly induce EVs secretion 89. This suggests that inflammasome activation may not be required for exosomal secretion.

Although most of the available references indicate that inflammasome activation induces exosomal secretion, the number of such studies is still limited. Due to the contradictory results, the direct effect of inflammasome activation on exosomal secretion remains inconclusive at this phase. Perhaps, types of PRRs, activators and the effector cells are important to determine the outcome. Further elucidations are required to address these issues.

### Effects of exosomes on inflammasome activation: Exosomes hinder inflammasome activation

Many recent studies have shown that the release of exosomes is a strategy of the cells to ease inflammation and to prevent tissue damage, which is a common end-point of excessive inflammatory response, by inhibiting inflammasome activation. Interestingly, most of these studies investigated exosomes derived from various stem cells (**Table 3**). As mentioned above that oxidative stress is one of the stimuli for NLRP3 inflammasome activation, exosomes released from BMSCs can diminish H_2_O_2_-induced inflammation and cell death in nucleus pulposus cells by reducing expression levels of NLRP3, caspase-1 and IL-1β, and decreasing cleavages of pro-caspase-3 and pro-caspase-9 16. The BMSCs-derived exosomes can also attenuate mitochondrial damage induced by H_2_O_2_, and injection of these exosomes into a rabbit model of intervertebral disc degeneration (IVDD) can delay the progression of IVDD 16. These inhibitory effects of BMSCs-derived exosomes may be driven by mitochondrial proteins carried by the exosomes. Because H_2_O_2_ causes oxidative stress and mitochondrial damage, providing the cells with fresh mitochondrial proteins by the exogenous exosomes therefore minimizes the damage and, as a consequence, reduces oxidative stress 16.

Doxorubicin is commonly used for cancer treatment; however, it has been shown to stimulate inflammatory response by inducing expression of TLR4, NLRP3, caspase-1, caspase-11, gasdermin D and IL-1β in cardiomyoblasts 96. By treating the cardiomyoblasts with exosomes derived from embryonic stem cells (ESCs), this inflammasome activating property of doxorubicin is minimized, whereas the exosomes derived from mouse embryonic fibroblasts have no effect on doxorubicin-induced upregulation of those inflammasome mediators 96. These differential effects of exosomes derived from different cell types indicate cell-specific cargo of biomolecules carried by exosomes.

Retinal inflammation is one of the pathologic findings of diabetic retinopathy. High levels of IL-1β, IL-18 and caspase-1 are detected in vitreous body of diabetic rats 97. In addition to the vitreous body, the inflammasome-related proteins, including NLRP3, IL-1β, IL-18 and caspase-1, increase in retina of diabetic rats and human retinal endothelial cells exposed to high glucose. This diabetes-induced inflammasome activation is abolished by treatment with exosomes secreted from hUCMSCs, but not the exosomes derived from dermal fibroblasts 97, again indicating the cell-specific exosomal cargo. Such injection of hUCMSCs-derived exosomes led to higher level of miR-126 in the retina, and pretreatment of the exosomes with miR-126 before injection results in dramatic induction of miR-126 in the retina 97. Such pretreatment is more effective to suppress NLRP3 inflammasome activation induced by high glucose, suggesting that miR-126 is responsible for the inhibitory effects of exosomes against inflammasome activation 97.

Similarly, other studies have shown that hUCMSCs-derived exosomes can reduce expression levels of NLRP3 and caspase-1 in LPS-treated macrophages and prevent IL-1β secretion from these cells 98, 99. These hUCMSCs-derived exosomes also decrease NLRP3 inflammasome in liver tissues of mice with acute liver injury 99. The intravenous injection of hUCMSCs-derived exosomes into rats with severe burn can reduce serum IL-1β level, and this anti-inflammatory effect of hUCMSCs-derived exosomes is most likely driven by miR-181c carried by these exosomes 98.

**Figure 3:**
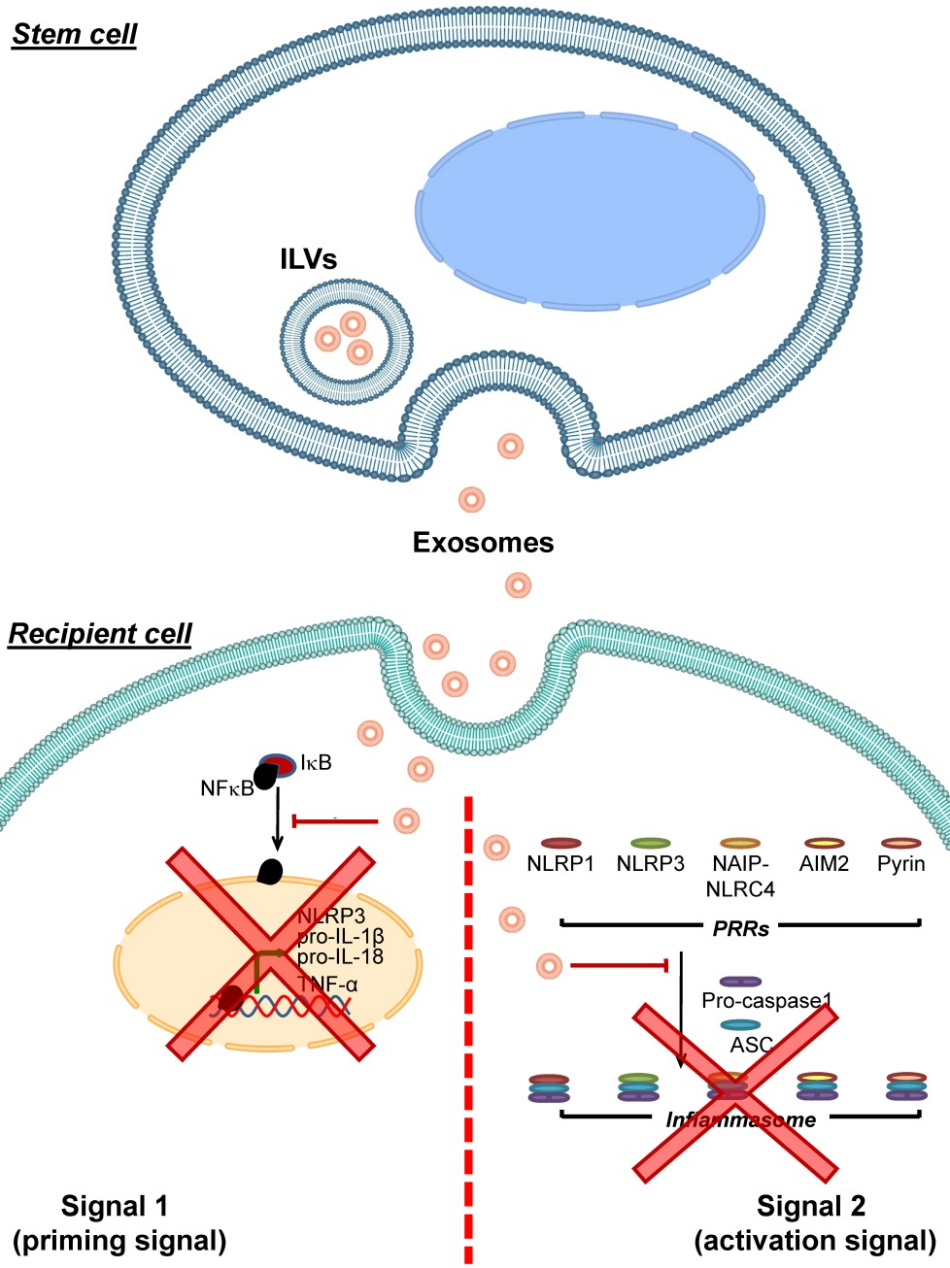
** Inhibition of inflammasome activation by stem cell-derived exosomes.** Exosomes released from various stem cells can be uptaken by recipient cells and then interfere with inflammasome activation. These stem cell-derived exosomes carry bioactive molecules (mainly miRNAs) that block the effects of signal 1 by preventing NF-κB dissociation from IκB, leading to inhibition of NF-κB nuclear translocation and reduced expression of its target genes encoding NLRP3, pro- IL-1β, pro-IL-18 and TNF-α. In addition, the stem cell-derived exosomes inhibit formation of the inflammasome complex by PRRs, ASC and pro-caspase-1. As a result, conversion of pro-caspase-1 to caspase-1 and secretion of the pro-inflammatory cytokines, particularly IL-1β, IL-18 and TNF-α, are reduced.

Adipose tissue-derived mesenchymal stem cells (AMSCs) have been shown to release exosomes containing high level of miR-17 100. These miR-17-containing exosomes can decrease levels of NLRP3, caspase-1, IL-1β and IL-18 in liver tissues of mice with acute liver failure. Introducing these miR-17-containing exosomes to macrophages or primary Kupffer cells alleviates LPS-induced inflammasome-related protein expression and IL-1β and IL-18 secretion in these immune cells. Moreover, blocking the miR-17 function hampers such inhibitory effects of these exosomes on inflammasome activation 100. A mixture of exosomes and microvesicles released from human periodontal ligament stem cells (hPDLSCs) obtained from relapsing-remitting multiple sclerosis patients are able to diminish TLR4, NF-κB, NLRP3, caspase-1 and IL-1β levels in spinal cords of mice with encephalomyelitis 101. All of these data indicate that exosomes derived from stem cells can inhibit inflammasome activation via various mechanisms (mainly by microRNAs carried by the exosomes) (**Figure 3**).

In addition to the MSCs-derived exosomes, periodontal ligament (PDL) cells, which are a component of periodontal tissue supporting teeth regularly exposed to mechanical force during mastication, are able to release exosomes 102. These exosomes can suppress NRLP3 activation and IL-1β secretion from LPS-primed and nigericin-treated macrophages by preventing NF-κB to translocate into the nucleus, thereby blocking the expression of NLRP3 and pro-IL-1β 102. EVs are also detected in fetal bovine serum (FBS), which is a common supplement in cell culture medium 103. These FBS-derived EVs can be uptaken by A549 lung epithelial cells and enhance their migration 103. Moreover, exosomes purified from FBS can diminish secretion of IL-1β from LPS-treated rat peritoneal macrophages 104. These data underscore the inhibitory roles of exosomes derived from various cells other than MSCS in modulation of the inflammasome activation. Because of the global interest in research on this topic, much more data will come in the coming years.

### Effects of exosomes on inflammasome activation: Exosomes enhance inflammasome activation

**Figure 4:**
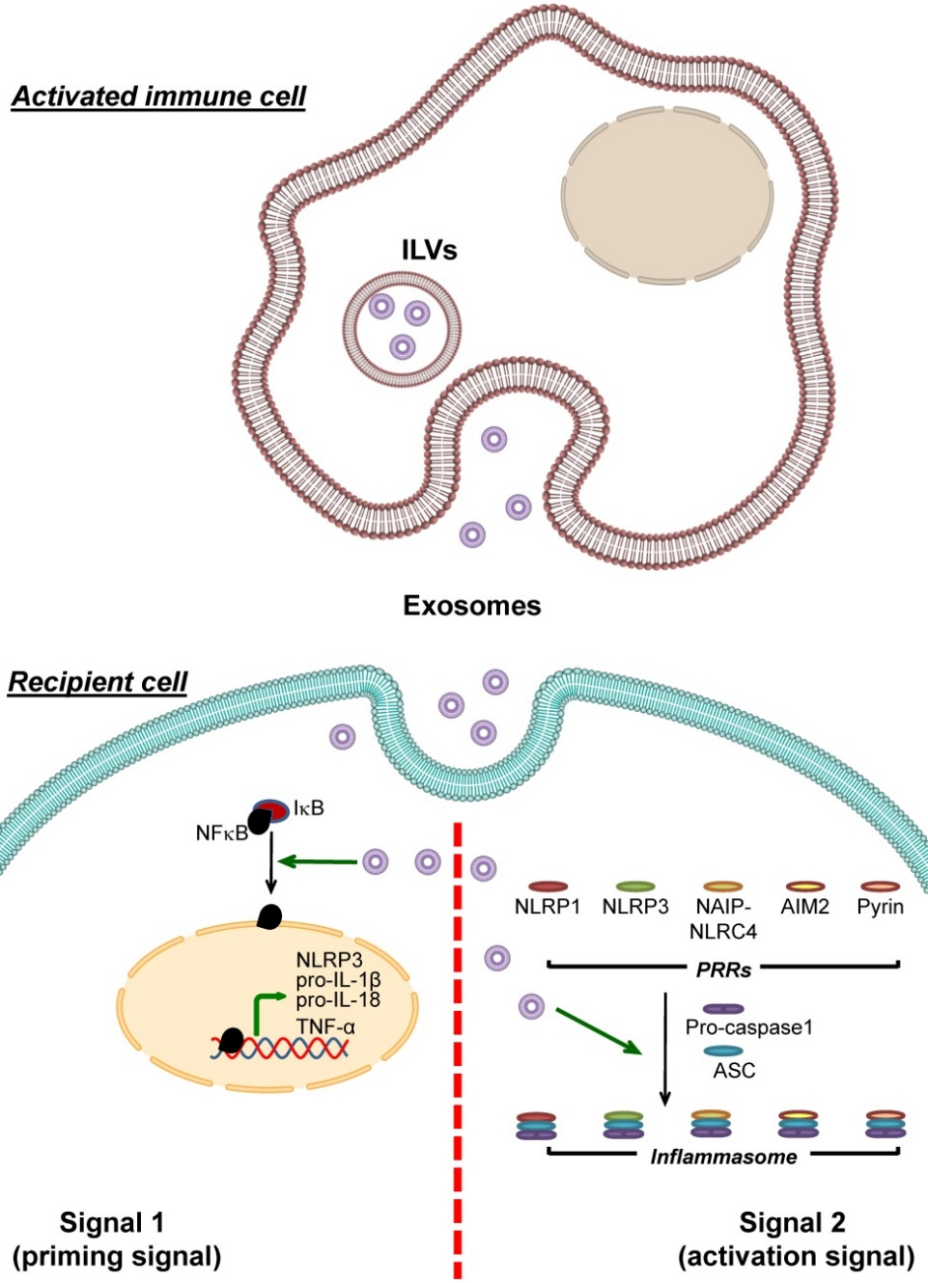
** Enhancement of inflammasome activation by immune cell-derived exosomes.** Exosomes derived from immune cells can be uptaken by recipient cells and then enhance inflammasome activation. These immune cell-derived exosomes carry bioactive molecules that enhance the effects of signal 1 by inducing NF-κB dissociation from IκB, leading to NF-κB nuclear translocation and upregulation of its target genes encoding NLRP3, pro- IL-1β, pro-IL-18 and TNF-α. In addition, the immune cell-derived exosomes enhance formation of the inflammasome complex by PRRs, ASC and pro-caspase-1. As a result, conversion of pro-caspase-1 to caspase-1 and secretion of the pro-inflammatory cytokines, particularly IL-1β, IL-18 and TNF-α, increases.

Unlike the exosomes derived from stem cells that negatively control inflammasome activation, the exosomes that activate inflammasome are originated from various cell types, including immune, cartilage, epithelial, and cancer cells (**Table 4**). Among these, immune cells-derived exosomes have the strongest evidence with the largest number of studies to support their roles in inflammasome activation (**Figure 4**).

Peripheral blood lymphocytes transfected with provirus plasmid of human immunodeficiency virus (HIV) can release exosomes containing an HIV protein namely Nef 73. Nef-containing exosomes purified from plasma of HIV-infected patients can be uptaken by the non-infected macrophages, resulting in enhancement of TLR4 translocation to the plasma membranes. As a consequence, these Nef-induced non-infected cells are prone to inflammatory stimuli and secrete more IL-1β and other inflammatory cytokines 105. ASC-containing EVs isolated from sera of traumatic brain injury patients upregulate expression of inflammasome components and promote inflammasome activation in human pulmonary endothelial cells 78.

BMDMs can secrete exosomes under stimulated and unstimulated conditions. The exosomes derived from BMDMs treated with nigericin following LPS priming can induce expression of NLRP3 and pro-IL-1β in naive BMDMs, and activate cleavage of pro-caspase-1 and secretion of IL-1β 106. Similarly, the resident immune cells like microglia are able to release exosomes, and the LPS-primed microglia exposed to manganese secrete greater amount of exosomes that possess ASC protein, a component of the inflammasome complex 71. When naive microgial cells are exposed to these ASC-containing exosomes, ASC can be transferred to the naive cells, leading to increases of NLRP3 and pro-IL-1β in the cells. Moreover, treatment of LPS-primed microglial cells with exosomes purified from sera of manganese-administered mice potentiates IL-1β secretion from the LPS-primed cells 71.

Other than the primary cultured immune cells, macrophage cell line, such as Raw264.7, can also secrete exosomes. Interestingly, the exosomes derived from LPS-treated Raw264.7 cells are uptaken by AML-12 hepatocytes and then upregulate expression of NLRP3, ASC and caspase-1 in the hepatocytes 75. Furthermore, injection of these exosomes derived from the LPS-treated Raw264.7 macrophages into mice can increase tissue levels of these inflammasome complex proteins *in vivo* 75.

In addition to the immune cells, other cell types can secrete exosomes that subsequently induce inflammasome activation. Exosomes derived from chondrocytes of patients with osteoarthritis (OA) are able to stimulate IL-1β secretion from LPS/ATP-treated phorbol-12-myristate-13-acetate (PMA)-induced THP1 macrophages 107. The exosomes derived from OA chondrocytes treated with IL-1β can induce ASC specks in the cytoplasm of the LPS-treated PMA-induced THP1 cells, indicating inflammasome activation. In addition, intra-articular injection of these exosomes accelerates cartilage erosion in a murine model of OA. RNA sequencing and bioinformatics analysis of miRNAs found in the exosomes derived from IL-1β-treated versus untreated OA chondrocytes reveal miR-449a-5p as a candidate molecule with great potential to elicit stimulatory effect on inflammasome activation. As such, treatment with a miR-449a-5p inhibitor effectively blocks this stimulatory effect of exosomes derived from the IL-1β-treated OA chondrocytes 107.

Furthermore, human adult retinal pigment epithelial (hARPE-19) cells irradiated by blue-light have been shown to express high levels of IL-1β, IL-18 and caspase-1, and secrete exosomes containing active forms of these inflammasome-related proteins 9. Incubation of the unirradiated hARPE-19 cells with these exosomes increase levels of NLRP3 mRNA/protein and active forms of IL-1β, IL-18 and caspase-1 9. A cancer cell line, such as HepG2, can secrete EVs in response to palmitate fatty acid treatment. The exosomes released from palmitate-treated HepG2 cells, but not those from vehicle-treated cells and HepG2 treated with palmitate plus lipid-lowering drug (ezetimibe), are able to increase pro-IL-1β expression and the release of mature IL-1β from THP-1 monocytic cells 108. Moreover, exosomes isolated from malignant ascites and amniotic fluid can induce pro-IL-1β expression and IL-1β secretion in THP-1 monocytic cells 57. These data obtained from both immune and non-immune cells highlight the important roles of exosomes for inflammasome activation.

## Roles of exosomes for non-inflammasome-mediated inflammation

Exosomes not only have an impact on inflammasome-mediated inflammation but also play roles in secretion of non-inflammasome-mediated inflammatory cytokines. Exosomes secreted by hUCMSCs contain miR-1246 and/or miR-181c 81, 98. These exosomes can reduce levels of pro-inflammatory cytokines, i.e., TNF-α, IL-6 and IL-17, in liver of mice with ischemia/reperfusion injury, liver of rats with severe burn, and LPS-treated macrophages 81, 98. In consistent, these hUCMSCs-derived exosomes increase levels of the anti-inflammatory cytokines, i.e., IL-10 and transforming growth factor β 81, 98. Exosomes derived from COM-treated macrophages are able to induce IL-8 secretion from renal tubular cells and monocytes 76, 77. Exosomes isolated from sera of the LPS-treated mice increase expression levels of TNF-α and IL-6 pro-inflammatory cytokines in lungs of the naive mice injected with these exosomes. These pro-inflammatory properties have been proven to be driven by miR-155 packed into these exosomes 70. Furthermore, exosomes purified from semen of the fertile men can stimulate secretion of pro-inflammatory cytokines, IL-6 and IL-8, from human endometrial stromal cells 17. By contrast, exosomes purified from plasma of patients with esophageal squamous cell carcinoma induce anti-inflammatory IL-10 expression in CD19^+^ B-cells isolated from healthy donors and inhibit proliferation of these cells 109. These findings indicate that exosomes also play significant roles in the non-inflammasome-mediated inflammation.

## Therapeutic roles of exosomes

Since exosomes are able to carry bioactive molecules from one cell to the others and subsequently cause changes in the recipient cells, much wider attention and more extensive efforts have been made recently to implement exosomes as the new therapeutic tools for delivering therapeutic molecules to modify inflammation and related diseases. Several lines of recent evidence have shown that exosomes can be used for delivering short‐interfering RNAs (siRNA) and miRNAs *in vivo*. An advantage of using exosomes as the carrier for these RNAs is that exosomes prevent RNAs degradation by RNase, thereby increasing the efficiency of RNAs delivery to the target tissues 110. For example, ASC siRNA can be loaded into exosomes prepared from culture medium of rat cortical neuronal cells 67. The injection of these ASC siRNA-loaded exosomes into circulatory system of rats with spinal cord injury reduces ASC level in the spinal cords, thereby reducing caspase-1 activation and IL-1β level in the spinal tissue 67.

Similarly, miR-181c found in exosomes secreted from hUCMSCs can reduce TLR4 expression and NFκB activation both *in vitro* and *in vivo* 98. As a consequence, secretion of pro-inflammatory cytokines decreases. Overexpression of miR-181c in hUCMSCs-derived exosomes strongly reduces white blood cell recruitment to the burn site and pro-inflammatory cytokine levels in the wound area in a rat model of severe burn 98. Administration of hUCMSCs-derived exosomes rich in miR-146a alleviates inflammation by decreasing TNF-α pro-inflammatory cytokine and increasing IL-10 anti-inflammatory cytokine in LPS-treated BMDMs, and improves survival rate of mice with sepsis 111. Moreover, exosomes derived from M2 polarized BMDMs containing miRNAs, particularly miR-690, rescue insulin sensitivity in Type 2 diabetic mice 112. Mechanistic study to define the molecule responsible for such therapeutic effect reveals miR-690 that exerts the therapeutic function to improve the insulin sensitivity by interfering with mitochondrial NAD^+^ kinase level 112. Similar approach reveals that exosomes with miR-22-3p upregulation can reduce LPS-induced acute lung injury by downregulating frizzled class receptor 6 (FZD6) protein 113. These data strongly suggest that exosomes can serve as the new therapeutic tools to modulate inflammatory responses in various diseases.

## Summary and outlook

Recent evidence has strongly indicated the crosstalk between exosome and inflammasome. On one hand, inflammasome activation can regulate the release of exosomes. Inflammasome can be activated by various stimuli and, as a result, exosomes released under different conditions, treatments or interventions may carry different components. However, precise mechanisms governing specific exosomal cargo loading are not fully understood. Moreover, number of the direct evidence for the effects of inflammasome activation on exosomal secretion is too small and the contradictory results make such effects inconclusive at this stage. Further elucidations on these aspects are therefore required.

On the other hand, exosomes are the upstream of inflammasome activation. Exosomes can either alleviate or enhance inflammasome activation. This discrepancy of exosomal effects on the inflammasome activation is likely affected by type of the cells producing exosomes and interventions or conditions that induce cells to release the exosomes. These two factors affect molecular contents of exosomes and hence determine the effects of exosomes on the target cells. Regardless of inhibitory or stimulatory effects of exosomes, miRNAs are the important molecules eliciting exosomal functions. According to the available references published to date, exosomes released from stem cells elicit inhibitory effects against inflammasome activation in the recipient cells, thereby reducing inflammatory response and preventing tissue damage caused by prolonged inflammation. By contrast, exosomes released from immune cells provoke inflammasome activation, leading to intensification of inflammation and inflammatory diseases. However, the information about the effects of exosomes on inflammasome function at molecular level is still insufficient. It is likely that exosomes released from different immune cells exert differential immunomodulatory activities via different compositions within the exosomal cargo.

During the past few years, a wide attention has been made to the therapeutic potential of exosomes. They can serve as the cargo to transfer the bioactive molecules that can be applied as the drug delivery system for treatment of various human diseases. However, number of the original investigations reporting the results is relatively small. Two questions raised here is the specificity of such therapeutic effects and adverse events of exosomes. A study in mice reveals distribution of the injected exosomes in liver, lung and spleen, but not in cardiac adipose tissue and skeleton muscle, suggesting differential abilities of different tissues to uptake the exogenous exosomes 114. This study also demonstrates that the injected exosomes are likely to be uptaken by various other cells nonspecifically. Nevertheless, this point is still far from conclusive. As discussed earlier, exosomes derived from different sources/cell types or from different conditions/treatments have different cargo compositions and properties. These differences probably affect exosomal endocytosis by the target cells and may make the process selective to some extent. For this reason, selection of the source of exosomes for a treatment should be done more carefully. In addition, management of different diseases/disorders with various target tissues may require exosomes from different sources. Almost all the studies reported so far have focused only on the ability of cells in the tissue of interest to uptake such exosomes and on the effects of the exogenous exosomes on that particular tissue. However, they have not investigated whether exosomes also exert their effects in other tissues. Moreover, whether inflammasome manipulation affects the therapeutic effects of the administered exosomes remains unclear. Therefore, these aspects deserve further elucidations. Finally, large-scale prospective clinical trials are required to investigate the specific therapeutic effects and adverse events of the exogenous exosomes before they can be applied to clinical practice.

## Figures and Tables

**Table 1:** Signal 1 of inflammasome activation system

Pattern recognition receptors (PRRs)	Activators
TLR	**PAMPs**
	- LPS 115, 116
	- Peptidoglycan 116
	- Zymosan 115, 117
	- Lipoproteins 118
	**DAMPs**
	- Heat shock proteins 119, 120
	- Histones 121
	- Defensins 122
IL-1R	IL-1β 123
TNFR	TNF-α 124

**Table 2:** Signal 2 of inflammasome activation system

Pattern recognition receptors (PRRs)	Activators	
	PAMPs	DAMPs
NLRP1	*Bacillus anthracis* lethal toxin 125	NA
NLRP3	- *Aeromonas hydrophila* aerolysin 42	- ATP and K^+^ efflux 29, 42, 127
	- Gramicidin 42	- Amyloid-β 43
	- Influenza A virus 59	- Calcium pyrophosphate dehydrate crystals 128
	-* Mycobacterium tuberculosis* protein, ESAT-6 126	- Monosodium urate crystals 128
	- Maitotoxin 127	
	- Nigericin 42, 127	
	- *Staphylococcus aureus* α-hemolysin 42	
	- Viral RNA 59	
NAIP-NLRC4	- Bacterial type III secretion systems 129	NA
	- Bacterial flagellin 35	
	- *Pseudomonas aeruginosa* 130	
	- *Salmonella typhimurium* 131	
AIM2	Microbial cytoplasmic dsDNA 28, 39, 132	Host cytoplasmic dsDNA 28, 39, 132
Pyrin	Bacterial toxin^#^ 44	NA

N/A: No available information. #Pyrin does not directly bind to bacterial toxin, but requires inactivation of Rho GTPase caused by the toxin 133.

**Table 3:** Summary for sources and responsible bioactive molecules of exosomes with inhibitory effects against inflammasome activation

Sources of exosomes	Releasing cell type	Target cell(s)/tissue(s)	Effector molecule(s)	Reference
**With paracrine effects**				
Adipose tissue-derived mesenchymal stem cells (AMSCs)	Stem cell	Macrophage and Kupffer cell	miR-17	100
Bone marrow derived stem cells (BMSCs)	Stem cell	Nucleus pulposus cell	Mitochondrial proteins	16
Embryonic stem cells (ESCs)	Stem cell	Cardiomyoblast	N/A	96
Human periodontal ligament stem cells (hPDLSCs) from patients with relapsing-remitting multiple sclerosis *	Stem cell	Spinal cord	N/A	101
Human umbilical cord-derived mesenchymal stem cells (hUCMSCs)	Stem cell	Retinal endothelial cell	miR-126	97
hUCMSCs	Stem cell	Macrophage	N/A	99
hUCMSCs	Stem cell	Macrophage	miR-181c	98
Cyclic stretch-stimulated periodontal ligament (PDL) cells	Fibroblast-like cell	Macrophage	N/A	102
**With paracrine and/or autocrine effects**				
Fetal bovine serum ^#^	N/A	Macrophage	N/A	104

*The mixture of exosomes and microvesicles was used in the study. #EVs were used in the study. N/A: No available information.

**Table 4:** Summary for sources and responsible bioactive molecules of exosomes that induce inflammasome activation

Sources of exosomes	Releasing cell type	Target cell(s)/tissue(s)	Effector molecule(s)	Reference
**With paracrine effects**				
Amniotic fluid and malignant ascites	N/A	Macrophage	N/A	57
IL-1β-treated osteoarthritic chondrocytes	Cartilage cell	Macrophage	miR-449a-5p	107
Lipopolysaccharide (LPS)-treated Raw264.7 macrophages	Immune cell	Hepatocyte	N/A	75
Palmitate-treated HepG2 cells^#^	Liver cancer cell	Macrophage	N/A	108
Plasma of HIV-infected patients	HIV-target cell (Immune cell)	Non-HIV infected macrophage	HIV protein Nef	105
Plasma of traumatic brain injury patients^#^	N/A	Pulmonary endothelial cells	Apoptosis associated speck-like protein containing a caspase recruitment domain (ASC)	78
**With paracrine and/or autocrine effects**				
Blue-light irradiated human adult retinal pigment epithelial (hARPE-19) cells	Epithelial cell	Non-irradiated retinal pigment epithelial cell	Active forms of IL-1β, IL-18, and caspase-1	9
LPS/nigericin-treated bone marrow-derived macrophages (BMDMs)	Immune cell	Naive or nigericin-treated BMDMs	N/A	106
Manganese/LPS-treated microglial cells	Immune cell	Naive microglial cell	Apoptosis associated speck-like protein containing a caspase recruitment domain (ASC)	71

#EVs were used in the study. N/A: No available information.
